# Schinzel-Giedion syndrome: communication, feeding and motor skills in 16 individuals

**DOI:** 10.1007/s10048-025-00846-3

**Published:** 2025-08-27

**Authors:** Lottie D. Morison, Nuala Summerfield, Dana Bradley, Bregje W. van Bon, Angela T. Morgan

**Affiliations:** 1 Speech and Language Team, Murdoch Children’s Research Institute, c/o 50 Flemington Road, Parkville, VIC 3052 Australia; 2 Schinzel-Giedion Syndrome Foundation, West Sussex, UK; 3 Department of Human Genetics, Radboud UMC, Nijmegen, the Netherlands; 4 Department of Paediatrics, The Royal Children’s Hospital, Parkville, VIC Australia; 5 Department of Audiology and Speech Pathology, The University of Melbourne Parkville, Melbourne, VIC Australia

**Keywords:** Schinzel-Giedion syndrome, *SETBP1*, Phenotype, Communication, Developmental and epileptic encephalopathy, Childhood dementia

## Abstract

Schinzel-Giedion Syndrome (SGS) is a rare neurodevelopmental disorder caused by pathogenic *SETBP1* gain-of-function variants. SGS medical features have been well described. Associated skills critical to quality of life have such as communication, feeding, and motor skills are yet to be characterised. Here we used standardised caregiver report tools to characterise these skills as well as the medical features, in 16 children with SGS (median = 5 years, 7 months, range 6 months to 12.5 years). Vineland-3 scores reflected severe impairment in communication, daily living, socialisation and motor skills. Average receptive and expressive language skills were equivalent to a 0-to-1-month-old. Average motor skills were slightly stronger with age equivalents of 2-months-old for gross motor skills and 4-months for fine motor skills. 13/16 (81%) children could attend to someone’s voice, and 15/16 (94%) could make happy vocalisations. One individual (6%) could follow basic instructions. Despite a relatively homogenous phenotype, some children presented with relative strengths when compared to the rest of the cohort. Our expanded phenotype of SGS allows better targeted therapies and supports, highlighting the importance of early feeding intervention and augmentative and alternative communication (e.g., assistive technology for communication). Given the severity of the SGS profile, our data highlight the need for sensitive measurement tools for detecting subtle skill changes in SGS in response to precision medicine interventions.

**Supplementary Information:**

The online version contains supplementary material available at 10.1007/s10048-025-00846-3.

## Introduction

**Fig. 1:**
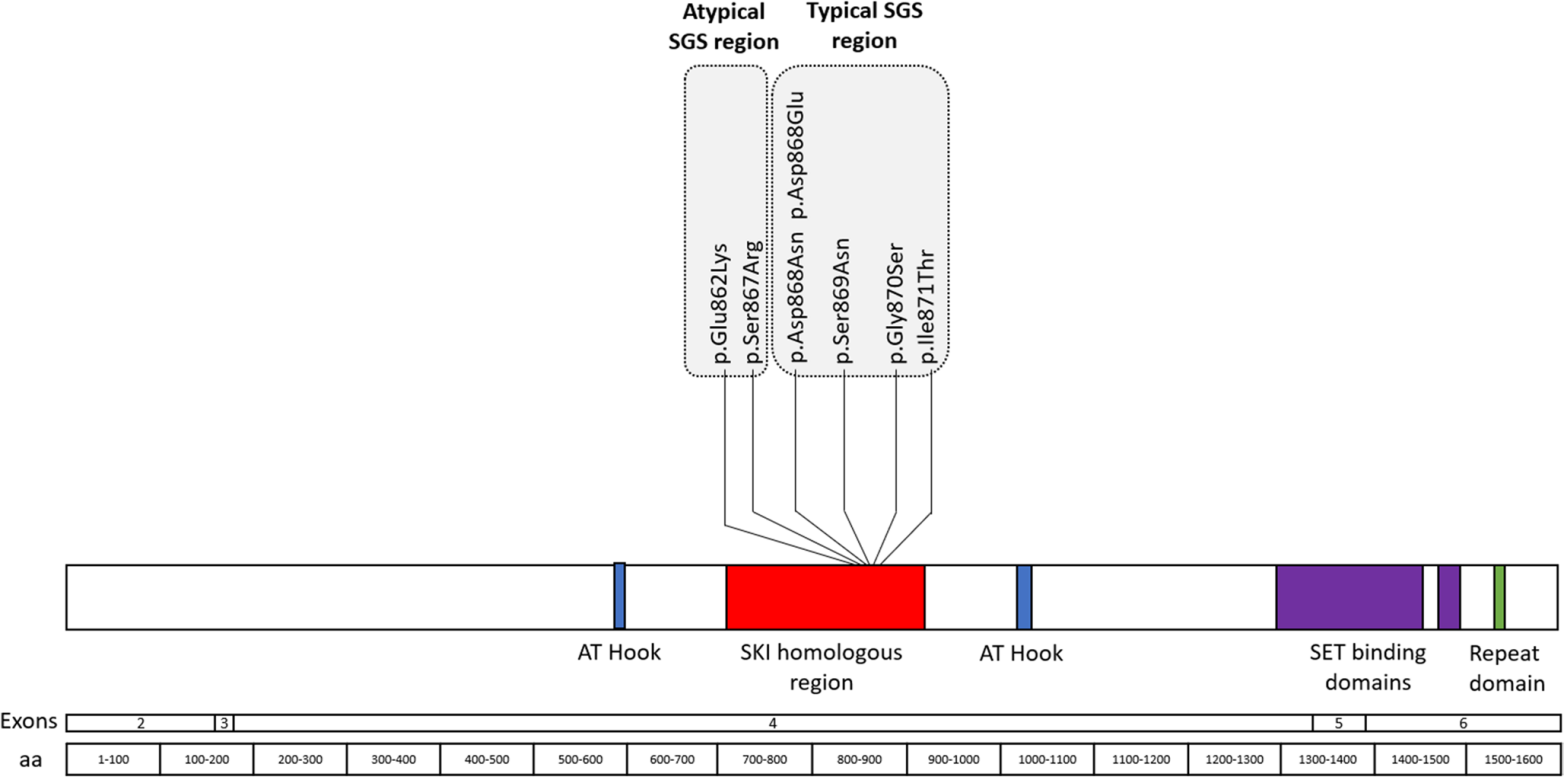
Seven heterozygous, missense, pathogenic, gain-of-function variants in SETBP1 in 16 individuals with Schinzel-Giedion Syndrome. The atypical Schinzel-Giedion Syndrome variants sit outside the 12 amino acid hot spot degron, whilst the typical Schinzel-Giedion Syndrome variants sit inside the 12 amino acid hot spot degron. (NM_015559.2)

Schinzel-Giedion Syndrome (SGS) is a neurodevelopmental condition caused by *SETBP1* gain-of-function variants [1]. SGS is a rare genetic disorder with most individuals with SGS passing away in the first decade of life [2–4]. Given a distinctive facial gestalt and clinical phenotype, individuals were described with this syndrome in the literature prior to *SETBP1* being identified as the causative gene at 18q12.3 [5–7]. Thirty-two years after the first characterisation of the SGS phenotype, *SETBP1* gain-of-function variants in the SKI homologous hot spot region in exon 4 were identified as explanatory for SGS [5, 8]. Pathogenic SGS variants are found in a 12-base pair hot spot region in the SETBP1 protein’s SKI domain (868aa-871aa, Fig. 1) [1, 8]. *SETBP1* codes for SET binding protein 1 (SETBP1), a DNA binding protein, which upregulates target genes at the level of transcription [9]. *SETBP1* variants within the hot spot region cause *SETBP1* gain-of-function and the classic SGS phenotype, and pathogenic variants just outside the hot spot cause atypical SGS [1]. Classic and atypical SGS have more involved phenotypes, with greater neurodevelopmental and medical implications, than *SETBP1* loss-of-function variants (known as *SETBP1*-haploinsufficency disorder) [10–12]. *SETBP1* variants have also been implicated in haematological malignancies [9].

*SETBP1* gain-of-function variants damage DNA in neural progenitor cells, ultimately causing neurodegeneration [13]. Electroencephalogram (EEG) and Magnetic Resonance Imaging (MRI) data also confirms the progressive neurological deterioration in the first few years of life [14, 15]. Despite a 40-year history in the medical literature, SGS remains under-described, with over 50 individuals with molecularly confirmed classic SGS, and seven individuals with atypical SGS published to date [1, 3, 4, 16–20].

The features of SGS are multifaceted, including impacts on cardiac, renal, musculoskeletal, urogenital, and neurological systems [1, 6]. Distinct facial features such as, a prominent forehead, low-set ears, mid-face retraction and orbital hypertelorism are seen [6]. Children also experience cortical visual impairment and severe sensorineural hearing loss [6]. Seizures are usually intractable, and MRI studies reveal clinically significant brain anomalies, such as hypoplasia of the corpus callosum, and atrophy of white matter connective pathways and the basal ganglia [15, 17, 21–24]. Neoplasia is common, especially of neuroepithelial origin [3, 25, 26]. Urinary tract infections are also common, due to hydronephrosis, and other renal malformations may occur [27]. Enteral feeding is required for many children with SGS due to severe oral feeding difficulties [28].

Given this complex medical background, development in SGS is severely to profoundly impaired [6, 16, 23, 25, 29]. Conversely to children with SGS, individuals with *SETBP1*-haploinsufficency disorder experience a far less medically involved phenotype, characterised by a severe speech movement disorder (typically childhood apraxia of speech with accompanying dysarthria in some), language disorder, mild to average cognitive ability and mild vision impairment [11, 12]. The phenotype of individuals with *SETBP1*-haploinsufficency disorder has been well characterised whereas the phenotype of SGS, particularly feeding, adaptive behaviour, motor and communication skills, remains comparatively obscured [1, 11]. Here we examine communication, feeding and motor skills in a cohort of children with SGS to inform prognosis and therapeutic decision making.

## Materials and methods

The SGS Foundation (https://sgsfoundation.org/) referred caregivers of children with clinically confirmed pathogenic or likely pathogenic *SETBP1* gain-of-function variants older than 6 months old to the study. Caregivers provided a genetic testing report to verify a child’s genetic diagnosis. Bereaved caregivers of children who had passed away in the last 6 years were also invited to participate. Caregivers provided written, informed consent for their child to participate in the study (Royal Children’s Hospital Human Ethics Committee, HREC 37353 A).

Caregivers completed a comprehensive medical questionnaire with the SGS foundation, through CombinedBrain (https://combinedbrain.org/). This questionnaire begins with a general health and development survey. Based on caregivers’ responses to this survey, they are directed to further questions about a given body system (e.g., vision, hearing, seizures). Caregivers completed this questionnaire on their own or via video call with a researcher trained in Human Subjects Research (DB). If caregivers completed the questionnaire on their own, responses were later verified during a video call.

A qualified speech pathologist (LDM) interviewed caregivers over video call on their child’s daily communication skills, current and previous therapies, and preferred activities, for example response to sensory stimulus and sensory play preferences. During this video call families also completed all other questionnaires listed below, aside from the medical questionnaire.

Bereaved caregivers completed all surveys based on their most recent memories of their child and provided video footage to verify assessment results.

As SGS is ultrarare, the assessment battery was designed to be conducted online to facilitate participation regardless of location. Additionally, childhood dementias like SGS are associated with high caregiver burden [2, 30]. Therefore, the assessment battery was designed to efficiently evaluate communication, feeding, motor skills, and adaptive behaviour without placing excessive time demands on families.

*Feeding*: Caregivers completed the Paediatric Assessment Scale for Severe Feeding Problems (PASSFP) [31]. The PASSFP is a 15-item validated, descriptive questionnaire which assesses children ≥ 1 year of age who may require enteral feeding [32]. The PASSFP has been validated for use in genetic disorders associated with severe feeding impairments [32]. The PASSFP provides a score from 0 to 66, with lower scores indicating more feeding difficulties.

*Adaptive behaviour and motor skills*: Caregivers completed the Vineland Adaptive Behaviour Scales 3rd Edition Comprehensive Caregiver Questionnaire (Vineland-3) to evaluate Communication, Daily living, Social skills and Motor skills [33]. The Vineland-3 Communication subdomains measure receptive, expressive and written language skills. Daily living and Socialisation skills are also each comprised of three subdomains. Daily living skill subdomains are caring for self, caring for home, living in the community, and socialisation subdomains are relating to others, play and leisure time and adapting. Motor skills has two subdomains: fine and gross motor skills. Communication, Daily living, and Social skills combine to provide an overall Adaptive Behaviour Composite score. The Vineland-3 provides standard scores for domains and *v*-scaled scores for subdomains, and age equivalents for subdomains.

The Vineland-3 is a multidimensional tool which measures several domains and has multiple scoring methods (e.g., age equivalents), which are better suited for individuals with severe impairments. Additionally, the Vineland-3 is already used in several rare diseases associated with conditions caused by SGS (e.g., motor impairment, visual impairment, epilepsy) [34–36].

*Communication*: Further to the Vineland-3 communication domain above, caregivers also completed the Communication Matrix [37]. The Communication Matrix is a descriptive assessment that assesses communication skills from pre-intentional communication such as crying in response to a stimulus (Level 1), through to symbolic communication such as combining spoken words or sign language to communicate (Level 7). The Communication Matrix stratifies these communication skills into four reasons to communicate: (i) to refuse, (ii) to obtain, (iii) for social reasons, and (iv) for information. In typical development, children achieve all seven levels of communication skills across the four reasons to communicate by 2-years-old. The Communication Matrix complemented the Vineland-3 as the Communication Matrix provides more descriptive information about communication, particularly for individuals with severe communication impairments. The Communication Matrix has also been used with individuals with genetic disorders and severe communication impairment, like Batten disease and Angelman syndrome, and is available in multiple languages [38, 39]. Caregivers also provided video footage of their child to verify results obtained from the Communication Matrix and Vineland-3. A simple linear regression analysed the association between participant age and Vineland-3 receptive and expressive subdomain age equivalent scores.

## Results

### Participants

Twenty-eight (*n* = 28) participants were referred to the study, 26 of whom were eligible to participate. Seven participants did not provide consent to participate in the study, and three participants consented but were subsequently lost to follow up. The two participants who were not eligible did not have *SETBP1* variants in or adjacent to the SGS 12 amino acid hot spot degron. Data was obtained for 16 participants (male = 9, female = 7) with pathogenic *SETBP1* missense variants (Fig. 1; Table 1). Participants were from the United States (*n =* 7), the United Kingdom (*n =* 4), one participant each from Argentina, Canada, Denmark, Hong Kong, and Serbia. Several participants shared the same missense variant, as depicted in Fig. 1. Participants 1–4 had atypical SGS with variants just outside the SGS degron (outside amino acid residues 868 to 871, Fig. 1; Table 1). Four participants had passed away between one (*n* = 2) and six years (*n* = 2) prior to the study, with their caregivers providing retrospective information on their child (Table 1). The median age for all 16 children was 5 years 7 months (Q1-Q3 = 1 year 10 months – 7 years 9 months), and the median age for the 12 living children was also 5 years 7 months (Q1-Q3 = 1 year 3 months − 8 years 1 month). Participant 2 also had a confirmed pathogenic compound heterozygote in *GJB2*, which is implicated in autosomal recessive hearing loss.

**Table 1:** Genotype and demographic information of 16 individuals with Schinzel-Giedion syndrome

Participant details			Genotype		Medical conditions					PASSFP score
Participant ID	Age at assessment (years)	Sex	c.DNA^†^	Protein	Visual impairment	Hearing impairment	Seizures	Cancer	Urogenital abnormalities	
1^‡^	10.2	F	c.2584G > A	p.Glu862Lys	+	-	+	-	+	45
2^‡^	6.53	M	c.2601 C > A	p.Ser867Arg	+	+	-	-	+	51
3^‡^	2.06	M	c.2601 C > G	p.Ser867Arg	+	-	+	-	-	44
4^‡^	12.52	F	c.2601 C > G	p.Ser867Arg	+	+	+	-	+	11
5	4.76*	M	c.2602G > A	p.Asp868Asn	+	+	+	-	+	9
6	7.83*	F	c.2602G > A	p.Asp868Asn	+	+	+	-	+	12
7	5.97	M	c.2604 C > T	p.Asp868Glu	+	-	+	-	+	30
8	0.65	M	c.2606G > A	p.Ser869Asn	+	+	+	-	+	TY
9	3.86*	M	c.2608G > A	p.Gly870Ser	+	-	+	+	+	7
10	1.13	M	c.2608G > A	p.Gly870Ser	?	+	+	-	+	35
11	7.43	F	c.2608G > A	p.Gly870Ser	+	-	+	-	-	46
12	10.16	F	c.2608G > A	p.Gly870Ser	+	-	+	-	+	10
13	7.84*	M	c.2608G > A	p.Gly870Ser	NA	NA	+	-	+	34
14	5.26	F	c.2612T > C	p.Ile871Thr	+	-	+	-	+	44
15	1.3	F	c.2612T > C	p.Ile871Thr	-	+	+	+	+	12
16	0.51	M	c.2612T > C	p.Ile871Thr	+	+	+	-	+	TY

* = age passed away, † = heterozygous, de novo, missense, pathogenic, NM_015559.2, ‡ = atypical Schinzel-Giedion Syndrome outside 12 amino acid hot spot degron. + = feature present, - = feature absent,?=caregiver unsure, MRI = magnetic resonance imaging, NA = not assessed, PASSFP = paediatric assessment scale of severe feeding problems, score out of a total of 66. Lower scores indicate lower feeding skills. TY = too young to be assessed.

### Medical

All participants completed the CombinedBrain medical survey (Table 1). Participants 5, 6, 9 and 13 had died (4/16, 25%) between the aged of 3 years, 10 months to 7 years, 10 months. Causes of death were cardiac arrest (participants 5 and 9) and seizures (participants 6 and 13).

Visual impairment was common (14/16, 88%) and in the remaining participants, 1/16 (6%) caregiver was unsure if their child had a visual impairment and participant 15 did not have a visual impairment. The nature of the visual impairment included cortical visual impairment (4/16, 25%), optic nerve atrophy (3/16, 19%) and myopia (3/16, 19%). Hearing impairment was less common (9/16, 56%). Participants 9 and 15 had cancer (2/16, 13%), both malignant tumours as the base of the spine. 14/16 (88%) participants had urogenital conditions and 13/16 (81%) had musculoskeletal conditions.

All participants had seizures apart from participant 2 who had atypical Schinzel-Giedion Syndrome outside 12 amino acid hot spot degron (15/16, 94%). Seizure onset was at 0 to 3 months (8/16, 50%), 4 to 7 months (2/16, 13%), 8 to 11 months (2/16, 13%), 2 years old (1/16, 6%, participant 7) and 7 years old (1/16, 6%, participant 1). Age at seizure onset was not specified for participant 5. 14/16 (88%) caregivers provided detailed information on additional neurological features. Hypotonia was more common (11/14, 79%) than hypertonia (9/14, 64%). 4/14 (29%) had encephalopathy.

### Feeding

Digestive issues were frequent (15/16, 94%), with only participant 3 unaffected. Details of digestive issues were provided by 14/16 (88%) participants, and included oesophageal (6/14, 43%), stomach (6/14, 43%), intestinal (6/14, 43%) and liver (1/14, 7%) concerns. 11/14 (79%) participants provided data on oral health, and several (6/11, 55%) participants had oral health concerns.

**Fig. 2:**
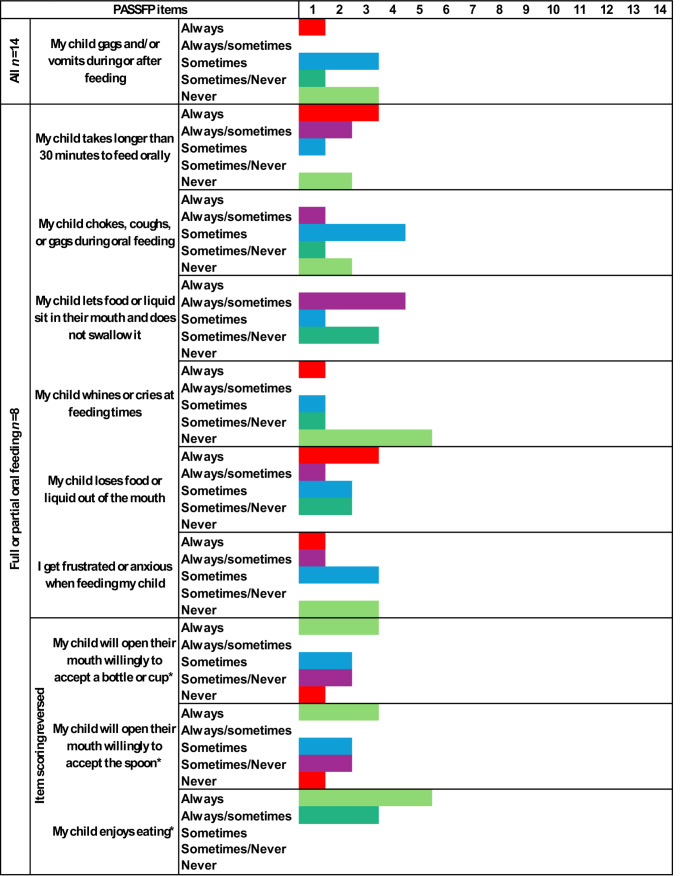
Feeding skills of 14 individuals with Schinzel-Giedion Syndrome children ≥ 1 year of age as measured by the Paediatric Assessment Scale for Severe Feeding Problems (PASSFP).* = items scoring reversed. Red = more severe feeding impairment, green = less severe feeding impairment

Fourteen caregivers completed the PASSFP (Fig. 2). 8/14 (57%) children had some food or drink orally (Fig. 2) with 3/8 (38%) children could only take small tastes of food. Most (9/14, 64%) relied on tube feeding for nutrition. One-quarter of children (4/14, 29%) ate orally with special modifications without enteral feeding, one child (1/14, 7%) primarily fed orally but had some supplemental enteral feeding. All children who could eat orally (8/8, 100%) could eat liquid and puree consistencies, preferably at room temperature. Participants 1 and 11 could eat foods of a variety of temperatures, and a range of consistencies; from liquids to soft chewable foods (e.g., potato chips). Participant 9 could also eat hard chewable foods (e.g., apples). Due to the open mouth position, dysmorphology that is characteristic of SGS [28], most children opened their mouth to accept a bottle or spoon (5/8, 63%). Children coughed, choked, or gagged ‘sometimes’ when eating orally (4/8, 50%) and many children ‘always’ had anterior spillage (3/8, 38%). Six (6/14, 43%) participants could have any food or drink orally. The average PASSFP score was 25 (SD = 14, ranging 7–43 out of possible total of 66, Table 1, Fig. 2).

### Adaptive behaviour and motor skills

Vineland-3 scores were severely impaired across all domains (standard score normative mean = 100, standard score normative SD = 15). Daily living skills (standard score median = 44, Q1-Q3 = 38–53) ranged from age equivalents of 0 months to 9 months old (Table 2). In terms of specific daily living skills, 4/16 (25%) individuals ‘sometimes’ cooperated with parents dressing and undressing them. Individuals could ‘sometimes’ (3/16, 19%) and ‘usually/often’ (2/16, 13%) cooperate with hand and face washing. Participant 3 could feed themselves with a spoon, and participant 1 could ‘sometimes’ do this (2/16,13%). Participant 3 could ‘sometimes’ drink from a cup (1/16, 6%).

**Table 2:** Age equivalent scores (years: months) on the vineland adaptive behaviour scales 3rd edition

Participant ID	Age at assessment (years)	Communication (years: months)			Daily Living (years: months)			Socialisation (years: months)			Motor (years: months)	
		Receptive Language	Expressive language	Written	Caring for self	Caring for others	Living in the community	Relating to others	Playing & using leisure time	Adapting	Fine	Gross
1	10.2	0:5	0:7	< 3:0	0:8	< 3:0	< 3:0	0:9	0:2	< 2:0	0:6	0:6
2	6.53	0:1	0:4	< 3:0	0:2	< 3:0	< 3:0	0:3	0:7	< 2:0	0:4	0:1
3	2.06	0:0	0:0	-	0:7	-	-	0:0	0:0	-	0:5	0:1
4	12.52	0:0	0:0	< 3:0	0:0	< 3:0	< 3:0	0:0	0:0	< 2:0	0:3	0:1
5	4.76*	0:0	0:0	< 3:0	0:0	< 3:0	< 3:0	0:0	0:5	< 2:0	0:3	0:0
6	7.83*	0:0	0:0	< 3:0	0:0	< 3:0	< 3:0	0:1	0:2	-	0:3	0:0
7	5.97	0:1	0:2	< 3:0	0:9	< 3:0	< 3:0	0:2	0:3	< 2:0	1:4	0:7
8	0.65	0:0	0:1	-	0:4	-	-	0:0	0:0	-	0:3	0:1
9	3.86*	0:0	0:0	< 3:0	0:0	< 3:0	< 3:0	0:0	0:0	< 2:0	0:1	0:1
10	1.13	0:0	0:0	-	0:0	-	-	0:0	0:0	-	0:0	0:0
11	7.43	0:0	0:0	< 3:0	0:7	< 3:0	< 3:0	0:0	0:0	< 2:0	0:0	0:0
12	10.16	0:0	0:3	< 3:0	0:0	< 3:0	< 3:0	0:1	0:4	< 2:0	0:3	0:3
13	7.84*	0:1	0:1	< 3:0	0:0	< 3:0	< 3:0	0:1	0:1	< 2:0	0:3	0:1
14	5.26	0:0	0:0	< 3:0	0:5	< 3:0	< 3:0	0:1	0:0	< 2:0	0:3	0:1
15	1.3	0:7	0:3	< 3:0	0:7	< 3:0	< 3:0	0:4	0:7	< 2:0	0:4	0:1
16	0.51	0:0	0:0	-	0:4	-	-	0:0	0:0	-	0:0	0:0

- = children < 3 years do not complete these subdomains, * = age passed away

**Fig. 3:**
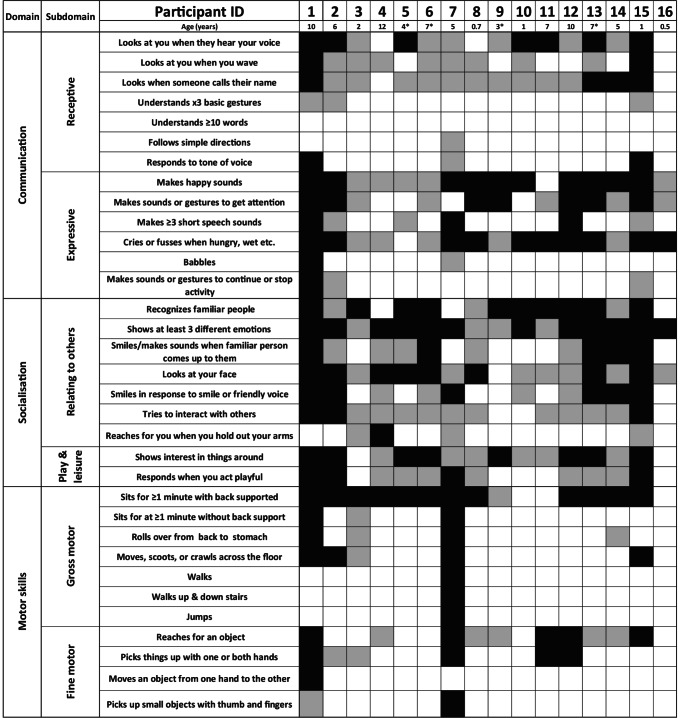
Key skills in 16 individuals with Schinzel-Giedion Syndrome as measured by the Vineland Adaptive Behaviour Scales 3rd Edition. Black shading indicates ‘usually/often’ and grey shading indicates ‘sometimes’. * = age passed away

Socialisation skills (standard score median = 32, Q1-Q3 = 28–45) ranged from 0 to 9 months old when relating to others, and 0 to 7 months old with play and leisure skills (Table 2). Half of the participants (8/16, 50%) had interpersonal and leisure skills at an age equivalent of 0-months-old. Caregivers identified that most children recognised familiar people (13/16, 81%), and all participants could exhibit at least three emotions such as happy, sad, surprised, afraid (16/16, 100%). Figure 3 illustrates commonly displayed Socialisation and Motor skills.

Motor skills mirrored Daily living and Socialisation (standard score median = 20, Q1-Q3 = 20–40), across fine (v-scale subdomain score median = 1, Q1-Q3 = 1–3) and gross motor skills (v-scale subdomain score median = 1, Q1-Q3 = 1–4, v-scale subdomain score normative mean = 15, v-scale subdomain normative SD = 3). Participant 7 was the only participant who could walk (16 months old age equivalent fine motor, 7 months old age equivalent gross motor), and five participants (5/16, 31%) could scoot across the floor using all four limbs (Fig. 3). Two children used a standing frame (2/16, 13%), Whilst motor skills ranged across the group, for most individuals gross motor skills were limited to sitting with back support. In terms of fine motor skills, some participants could reach for objects (10/16, 63%) and pick up objects (6/16, 37%). Across the group, average age equivalents were 4 months old for fine motor skills (age equivalent SD = 4 months), and 2 months for gross motor skills (age equivalent SD = 2 months). Overall, the Adaptive Behaviour Composite scores across the group were also low (standard score median = 39, Q1-Q3 = 32–48).

### Communication

Communication skills on the Vineland-3 were also severely impaired (*n* = 16, standard score median = 25, Q1-Q3 = 20–37, standard score normative mean = 100, standard score normative SD = 15), with most individuals having receptive and expressive language age equivalents to a 0- to 1-month-old (Table 2). Some participants exhibited receptive (v-scale subdomain score median = 1, Q1-Q3 = 1–3, v-scale subdomain score normative mean = 15, v-scale subdomain score normative SD = 3) and expressive (v-scale subdomain score median = 1, Q1-Q3 = 1–2) language skills equivalent to a 7-month-old child (Participant 1, Table 2). Average age equivalents for expressive and receptive language were 1-month-old (both age equivalent SD = 2 months). Participant age and age equivalent scores on receptive (*p* = 0.95, R^2^ = 0.00033, CI −0.32 to 0.30) and expressive (*p* = 0.22, R^2^ = 0.11, CI −0.12 to 0.47) subdomains were not associated.

Many children (13/16, 81%) could attend to someone’s voice and make happy sounds (15/16, 94%, Fig. 3). Some children would respond to their name (14/16, 88%). Participant 7 could follow simple directions, and 3/16 (19%) children responded to tone of voice (e.g., an angry or a happy tone of voice).

**Fig. 4:**
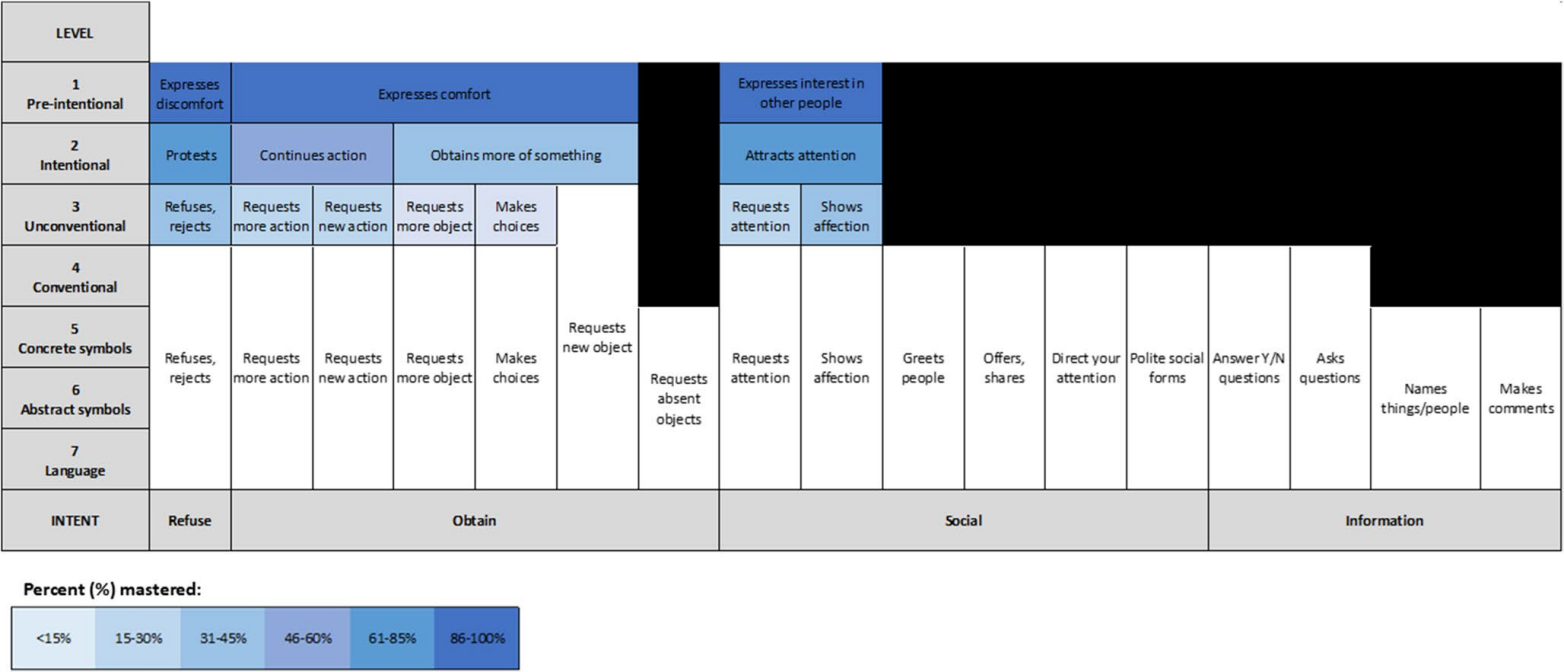
Communication skills of 16 individuals with Schinzel-Giedion Syndrome as measured by the Communication Matrix. Y/N = Yes/No

On the Communication Matrix (*n* = 16), all children used pre-intentional communication (e.g., Level 1, caregivers interpret an individual’s behaviours) to express discomfort, comfort, and interest in other people (Fig. 4). Some children could communicate intentionally (Level 2). The average percept of Communication Matrix skills mastered by the group was low (mean = 9%, SD = 4%). Refusing was a relative strength (mean skills mastered = 30%, SD = 10%), followed by communication for obtaining (mean skills mastered = 8%, SD = 5%) and social reasons (mean skills mastered = 8.28%, SD = 4%). The highest level an individual communicated at was Level 3 (unconventional behaviours, Fig. 3) and no participants could communicate for information reasons (e.g., answering yes/no questions, making comments). Participants 12 and 15 were the only participants who could request more of an object, and Participant 10 could make choices (e.g., purposefully reaching towards preferred item). Participants had a range of communication skills in their repertoires, with some individuals having > 10 methods to communicate refusal or to obtain (e.g., head, body and limb movements, facial expressions and vocalisations such as crying and screaming,), whilst others had fewer methods (i.e., three skills including whole body movement) to communicate for these reasons (Supplemental Table 1).

Eight participants were currently receiving speech therapy (8/16, 50%), primarily focussing on feeding skills, such as latching and oral stimulation for feeding purposes. Participant 2 had trialled an object-picture reference system (e.g., a spoon connected to a picture of eating to indicate food/mealtimes), and a high contrast yes/no choice board, although parents reported that neither of these systems were used successfully. Two participants (2/16, 13%) used switches for cause-and-effect play, such as activating a toy with lights. Some participants (3/6, 19%) had vision therapy, which included looking at high contrast black-and-white patterns, tracking objects and exposure to coloured lights. Two caregivers commented that their child could become overwhelmed if noise and visual stimulus (e.g., lights) were presented together, and appeared to prefer either noise or visual stimulus in isolation. For sensory play preferences, seven participants (7/16, 44%) enjoyed physical touch, and six participants enjoyed music (6/16, 38%).

In terms of loss of communication skills, some caregivers (5/16, 21%) commented that their child’s interaction with others had declined over time, including previously smiling (2/16, 13%) and humming and vocalising (1/16, 6%) with eventual loss of these skills. Several caregivers (8/16, 50%) observed that their child’s skills markedly fluctuated in response to seizures and general health and worsened over time.

## Discussion

SGS leads to a severe cognitive, language, motor and medical features. Here we have defined communication, motor, and feeding skills for the first time in classic and atypical SGS.

In this cohort, the four participants with atypical SGS exhibited phenotypes more in line with SGS than *SETBP1*-haploinsuffiency disorder, but had slight phenotypic differences to most participants with classic SGS [1, 40]. For instance, two participants with atypical SGS (participants 1, 4) were older at age 10 and 12 years, than most previously reported individuals with SGS [1]. Although there were still individuals with typical SGS variants of similar ages in our cohort, such as participant 12 (aged 10), and survival into adolescence has been previously reported in typical SGS [41]. Participants 2, 3 and 4 shared the same variant (p.Ser867Arg) just outside the degron as the individuals reported by Carvalho and colleagues [42]. Participants 1 and 7 presented with a slightly milder phenotype, with stronger skills across communication (participant 7 > 2 SD) and motor skills (participants 1 and 7 > 2 SD) compared to the rest of the cohort. Participant 7’s variant was within the classic *SETBP1* hot spot region, p.Asp868Glu. Yet, participant 7 exhibited marked strengths across Communication, Socialisation and Motor domains, being the only participant to follow simple directions, walk and jump. Participant 1’s variant was outside of the classic *SETBP1* hot spot region at p.Glu862Lys.

Seventeen individuals with SGS have been previously published with p.Asp868 variants with different amino acid substitutions (p.Asp868Asn *n* = 15; p.Asp868Ala, *n* = 1; p.Asp868Tyr, *n* = 1) [3, 43]. Variants in p.Asp868 lead to a large increase in SETBP1 protein levels, with an increased risk of cancer in comparison to other SGS variants [3, 25]. Whilst our participants with p.Asp868 variants (participants 5 and 6) did not have cancer they did pass away prior to 7 years of age. Participant 7 is the only reported individual with glutamate change at p.Asp868, with most previously reported cases at this location having an asparagine change. Yet, despite the location of participant 7’s variant, he had a relatively mild SGS phenotype at 5-years-old albeit still with significant impairments. Participant 7’s variant suggests a possible functional effect of these two amino acid changes; however, more cases are required to draw definitive conclusions. Hence, participant 7 and the individuals with atypical SGS variants reflects the variability of the SGS phenotype and warrants further investigation to elucidate the phenotypic heterogeneity.

Recently, SGS was identified as a promising candidate for gene therapy given a range of factors including the life-limiting nature of the condition and consequently a high-level of acceptable risk, the fact that several individuals share the same gain-of-function variants so that one targeted treatment could benefit many, and the existence of a coordinated, international support group (https://sgsfoundation.org/) to assist with participant recruitment [44]. Assessment of communication, adaptive behaviour and motor skills in genetic conditions can reflect responses to treatments, such as gene and enzyme replacement therapy [45]. Yet measuring communication and motor skills in SGS remains challenging given that many current tools are not sensitive enough for most children, exhibiting a floor effect. For instance, most of this cohort had an equivalent age of 0 years, 0 months on the Vineland-3 communication subdomains, which may result in a failure to capture any meaningful change between individuals or within individuals during the time course of an intervention. The subtly of communication and motor skills in individuals with SGS, alongside the impact of medical conditions (e.g., seizures), also requires the use of caregiver-reported assessments and subtle interpretations from caregivers often used with individuals with profound and multiple disabilities [46]. Yet descriptive caregiver assessments such as the Communication Matrix, PASSFP and item level analysis of the Vineland-3 used in this study provide further details of subtle skills and differences between individuals that may not be reflected through quantitative scores alone. In future, the utility of additional, descriptive caregiver report measures for use in SGS should be explored.

Knowledge about the communication, motor and medical features of SGS can inform provision and timing of therapies including speech, occupational, physio, and vision therapy. Whilst communication and motor skills were limited across the group, some children were learning alternative methods of communication. For instance, two children were learning to use switch technology (i.e., activating a button to create cause and effect). Switches are commonly used communication aid (also known as augmentative and alternative communication or AAC) with individuals with complex communication needs [47–49]. Other communication aids used with individuals with cognitive and vision impairment include tangible 3D symbols, which can support expression and comprehension particularly for those with vision and hearing impairment like in SGS [46, 47]. Communication intervention options for individuals with complex communication needs are not limited to communication aids [48]. Caregivers of children with severe communication impairment report benefits from communication partner training to feel empowered to see themselves as instrumental in their child’s communication and connect with other caregivers with similar experiences [50–52]. Likewise, intensive interaction techniques for individuals with severe intellectual disability, such as responsive vocal mirroring and joint focus activities, could support relationship building between individuals with SGS and their caregivers [53, 54]. In this cohort, many children responded to another person’s voice. Consequently, intensive interaction techniques such as vocal mirroring may be applicable for individuals with SGS [54]. Alongside communication supports, multisensory interventions using lights, sound and touch could act to reduce anxiety and challenging behaviours [55]. Characterising communication and motor skills in SGS allows for the identification of potential supports and therapies to inform the clinical decision making of clinicians, such as speech language pathologists’ decision making around communication aids for individuals with SGS [56]. The present study was cross-sectional. Longitudinal data is still required to inform the natural history of the syndrome and elucidate phenotype correlations in disease progression to inform future trials.

There are several limitations of the present study. Firstly, generalisations of results to the broader population of individuals with SGS should be approached with caution due to the small sample size. We also found that whilst 28 individuals were consented, only 16 participants completed the study. Hence, future SGS research should consider this and account for the high caregiver burden of individuals with SGS during recruitment and data collection. Secondly, small cohort size, and range of ages and variants inhibited analysis of genotype-phenotype correlations. Lastly, future studies should consider additional outcome measures that capture subtle communication and motor skills in SGS, as many individuals experienced a floor effect. Item analysis, as done here with the Vineland-3, could also prove useful. Additionally, use of objective and clinician-reported measures should be used, alongside caregiver report.

In conclusion, here we have provided important phenotypic data to enable better targeted therapies, guide prognosis and inform selection of outcome measures for future precision medicine trials.

## Supplementary Information

Below is the link to the electronic supplementary material.

Supplementary Material Supplementary information is available at Journal of Human Genetics’ website. **Supplemental Table 1.** Reason pattern scores and communication skills on the Communication Matrix. 1

## Data Availability

The data from this study is available upon reasonable request to the corresponding author.
