# Supplementary material for: Schinzel-Giedion syndrome: communication, feeding and motor skills in 16 individuals
Source: Neurogenetics. 2025 Aug 27;26(1):64. doi: 10.1007/s10048-025-00846-3 (PMC12380911; doi:10.1007/s10048-025-00846-3)
Supplement: Supplementary file 1 — Supplementary Material Supplementary information is available at Journal of Human Genetics’ website. Supplemental Table 1. Reason pattern scores and communication skills on the Communication Matrix. 1 [file 10048_2025_846_MOESM1_ESM.pdf]

Morison, L.D., Summerfield, N., Bradley, D., van Bon, B.W., Morgan, A.T. Schinzel-Giedion syndrome: Communication, feeding and motor skills in 16 individuals.

Neurogenetics.

Supplemental Table 1. Reason pattern scores and communication skills on the Communication Matrix

Participant ID	Reason pattern score*	Refuse	Obtain	Social
1	29:10:7:0	Arm movements<br>Change in posture<br>Coo<br>Cry<br>Fuss<br>Grunt<br>Grimace<br>Head movements<br>Limb movements<br>Scream<br>Smile<br>Squeal<br>Whine	Change in posture<br>Coo<br>Head movements<br>Look at person<br>Smile<br>Squeal	Change in posture<br>Coo<br>Fuss<br>Limb movements<br>Smile
2	29:10:7:0	Arm movements<br>Change in posture<br>Close mouth<br>Cry<br>Grunt<br>Head movements<br>Limb movements<br>Scream	Change in posture<br>Coo<br>Giggle<br>Open and close mouth<br>Smile<br>Squeal	Coo<br>Fuss<br>Smile<br>Squeal
3	14:3:4:0	Cry<br>Grunt<br>Head movements<br>Limb movements<br>Scream	Smile	Smile
4	43:10:14:0	Arm movements<br>Change in posture<br>Frown	Change in posture<br>Coo<br>Fuss	Arm/hand movements<br>Change in posture<br>Coo

Corresponding author: Angela T Morgan, angela.morgan@mcri.edu.au, Murdoch Children's Research Institute, c/o 50 Flemington Road, Parkville, Victoria, 3052, Australia

		Fuss<br>Grimace<br>Head movements<br>Limb movements<br>Scream<br>Whine	Head movements<br>Leg movements<br>Limb movements<br>Smile<br>Squeal	Fuss<br>Limb movements<br>Looks at you<br>Reaches foot towards you<br>Smile<br>Squeal<br>Touches you
5	14:3:4:0	Change in posture<br>Cry<br>Grimace<br>Grunt<br>Limb movements<br>Scream	Change in posture<br>Coo<br>Squeal	Change in posture<br>Eyes search for person
6	29:10:7:0	Arm movements<br>Change in posture<br>Close eyes<br>Cry<br>Fuss<br>Grimace<br>Grunt<br>Head movements<br>Leg movements<br>Limb movements<br>Scream<br>Whine	Arm movements<br>Change in posture<br>Head movements<br>Look at desired item<br>Look at person<br>Smile	Smile<br>Turn head<br>Wriggle
7	29:10:7:0	Arm movements<br>Change in posture<br>Cry<br>Frown<br>Fuss<br>Grimace<br>Grunt<br>Head movements	Approaches desired object<br>Arm movements<br>Bangs Table<br>Change in posture<br>Claps<br>Comes to you<br>Coo<br>Head movements	Approaches people<br>Head banging<br>Head movement<br>Smile

Corresponding author: Angela T Morgan, angela.morgan@mcri.edu.au, Murdoch Children's Research Institute, c/o 50 Flemington Road, Parkville, Victoria, 3052, Australia

		Leg movements<br>Limb movements<br>Move away from person or object<br>Scream<br>Whine	Holds hand<br>Limb movements<br>Look at person<br>Looks at desired item<br>PECS block for 'more'<br>Rocks<br>Smile<br>Squeal<br>Takes desired item	
8	29:3:7:0	Change in posture<br>Cry<br>Fuss<br>Grunt<br>Head movements<br>Leg movements<br>Limb movements<br>Scream<br>Whine	Change in posture<br>Coo<br>Limb movements<br>Squeal	Coo<br>Fuss<br>Squeal
9	43:10:14:0	Change in posture<br>Cry<br>Grunt<br>Head movements<br>Leg movements<br>Limb movements<br>Scream	Change in posture<br>Coo<br>Head movements<br>Leg movements<br>Limb movements<br>Squeal<br>Touches	Coo<br>Fuss<br>Limb movements<br>Squeal<br>Touches<br>Turns head
10	14:3:4:0	Cry<br>Grunt<br>Head movements<br>Limb movements<br>Scream	Coo<br>Holds finger<br>Less crying<br>Limb movements<br>Squeal	Change in posture
11	29:6:7:0	Change in posture<br>Cry<br>Frown	Arm movements<br>Change in posture<br>Facial expression	Change in posture<br>Coo

		Fuss<br>Grimace<br>Grunt<br>Leg movements<br>Limb movements<br>Scream<br>Spits out food<br>Whine		Fuss<br>Squeal
12	43:19:14:0	Arm or hand movements<br>Frown<br>Fuss<br>Grimace<br>Head movement<br>Head movements<br>Scream<br>Whine<br>Whole body movement	Activates switch to turn on toy piano<br>Approaches desired object<br>Arm/hand movements<br>Coo<br>Fuss<br>Head movement<br>Head movements<br>Leg movement<br>Leg movements<br>Moves head towards desired item<br>Reaches towards, touches or taps desired item (without taking it)<br>Squeal<br>Whole body movement (bounce up and down)<br>Whole body movement (lunge at object)	Arm/hand movements<br>Coo<br>Fuss<br>Smile<br>Squishes parents hands with her fingers if they put their hand in her fist. Expression is relaxed.<br>Squeal
13	29:6:7:0	Arm movements<br>Change in posture<br>Cry<br>Frown<br>Fuss	Arm movement<br>Change in posture<br>Coo<br>Head movements<br>Reach	Coo<br>Facial expression<br>Fuss<br>Squeal<br>Smile

Corresponding author: Angela T Morgan, angela.morgan@mcri.edu.au, Murdoch Children's Research Institute, c/o 50 Flemington Road, Parkville, Victoria, 3052, Australia

		Grimace<br>Grunt<br>Head movements<br>Scream<br>Spits out food<br>Whine	Squeal<br>Smile	
14	43:10:10:0	Change in posture<br>Cry<br>Fuss<br>Grimace<br>Grunt<br>Head movements<br>Limb movements<br>Scream<br>Stiffens<br>Vocalisations<br>Whine	Change in posture<br>Coo<br>Facial expression<br>Head movements<br>Limb movements<br>Look at person<br>Looks at desired object<br>Quiet<br>Squeal<br>Smile	Change in posture<br>Coo<br>Laugh<br>Looks at you<br>Squeal<br>Turn head
15	43:19:14:0	Close eyes<br>Leg movement<br>Whole body movement	Arm/hand movements<br>Looks at desired object<br>Tried to press button on toys	Coo<br>Giggles<br>Head movement<br>Head and leg movements<br>Smile<br>Smiles<br>Squeal<br>Vocalises
16	29:3:3:0	Arm movements<br>Change in posture<br>Cry<br>Fuss<br>Grimace<br>Grunt<br>Head movements	Change in posture<br>Facial expression	Change in posture<br>Coo<br>Fuss

Corresponding author: Angela T Morgan, angela.morgan@mcri.edu.au, Murdoch Children's Research Institute, c/o 50 Flemington Road, Parkville, Victoria, 3052, Australia

		Limb movements<br>Scream		

* = Reason pattern score as measured by the Communication Matrix, percent skills mastered across four reasons to communication

refuse:obtain:social:information
